# Brain region volumes and their relationship with disability progression and cognitive function in primary progressive multiple sclerosis

**DOI:** 10.1002/brb3.2044

**Published:** 2021-01-23

**Authors:** Francisco Carlos Pérez‐Miralles, Daniel Prefasi, Antonio García‐Merino, José Ramón Ara, Guillermo Izquierdo, Virginia Meca‐Lallana, Francisco Gascón‐Giménez, María Luisa Martínez‐Ginés, Lluis Ramió‐Torrentà, Lucienne Costa‐Frossard, Óscar Fernández, Sara Moreno‐García, Jorge Maurino, Joan Carreres‐Polo, Bonaventura Casanova

**Affiliations:** ^1^ Neuroimmunology Unit Department of Neurology Hospital Universitari i Politècnic La Fe Valencia Spain; ^2^ Department of Medical Roche Farma S.A Madrid Spain; ^3^ Department of Neurology Hospital Universitario Puerta de Hierro Majadahonda Spain; ^4^ Department of Neurology Hospital Universitario Miguel Servet Zaragoza Spain; ^5^ Department of Neurology Hospital Universitario Virgen Macarena Seville Spain; ^6^ Department of Neurology Hospital Universitario La Princesa Madrid Spain; ^7^ Department of Neurology Hospital Clínico Universitario de Valencia Valencia Spain; ^8^ Department of Neurology Hospital Universitario Gregorio Marañón Madrid Spain; ^9^ Girona Neuroimmunology and Multiple Sclerosis Unit Department of Neurology Hospital Universitari Josep Trueta and Hospital Santa Caterina IDIBGI Department of Medical Sciences Faculty of Medicine University of Girona Girona Spain; ^10^ Department of Neurology Hospital Universitario Ramón y Cajal Madrid Spain; ^11^ Department of Neurology Hospital Regional Universitario Carlos Haya Málaga Spain; ^12^ Department of Neurology Hospital Universitario 12 de Octubre Madrid Spain; ^13^ Department of Radiology Hospital Universitari i Politècnic La Fe Valencia Spain

**Keywords:** brain volume, cognitive function, disability progression, magnetic resonance imaging, primary progressive multiple sclerosis

## Abstract

**Background and purpose:**

Evidence on regional changes resulting from neurodegenerative processes underlying primary progressive multiple sclerosis (PPMS) is still limited. We assessed brain region volumes and their relationship with disability progression and cognitive function in PPMS patients.

**Methods:**

This was an MRI analysis of 43 patients from the prospective Understanding Primary Progressive Multiple Sclerosis (UPPMS) cohort study. MRI scans were performed within 3 months before enrollment and at month 12.

**Results:**

Gray matter volume of declive and white matter volumes adjacent to left straight gyrus, right calcarine sulcus, and right inferior occipital gyrus significantly decreased from baseline to month 12. Baseline white matter volumes adjacent to right amygdala and left cuneus significantly differed between patients with and without disability progression, as well as baseline gray matter volumes of left cuneus, right parahippocampal gyrus, right insula, left superior frontal gyrus, declive, right inferior temporal gyrus, right superior temporal gyrus (pole), and right calcarine sulcus. Baseline gray matter volumes of right cuneus and right superior temporal gyrus positively correlated with 12‐month Selective Reminding Test and Word List Generation performance, respectively. Gray matter changes in right superior semilunar lobe and white matter adjacent to left declive and right cerebellar tonsil also positively correlated with Word List Generation scores, while white matter change in left inferior semilunar lobe positively correlated with Symbol Digit Modalities Test performance after 12 months.

**Conclusions:**

White and gray matter volumes of specific brain regions could predict disability progression and cognitive performance of PPMS patients after one year.

## Graphical abstract

The data described in the present manuscript provide a detailed description of region volume changes exhibited by primary progressive multiple sclerosis patients over one year, including decreases in gray matter declive and white matter adjacent to left straight gyrus, right calcarine sulcus, and right inferior occipital gyrus. In addition, baseline volumes of white matter adjacent to right amygdala and left cuneus could also predict 1‐year disability progression in patients with primary progressive multiple sclerosis, as well as baseline grey matter volumes of left cuneus, right parahippocampal gyrus, right insula, left superior frontal gyrus, declive, right inferior temporal gyrus, right superior temporal gyrus (pole), and right calcarine sulcus. Furthermore, baseline volumes of certain regions such as grey matter of right cuneus and right superior temporal gyrus could also predict cognitive performance after one year, as well as volume changes in grey matter of right superior semilunar lobe and white matter of left inferior semilunar lobe and adjacent to left declive and right cerebellar tonsil were correlates of cognitive performance.

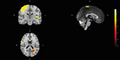

## 1 INTRODUCTION

Neurological processes underlying primary progressive multiple sclerosis (PPMS) entail central nervous system changes that may be evident in magnetic resonance imaging (MRI). Although PPMS patients usually have fewer brain T2 lesions and gadolinium‐enhanced T1 lesions than those with relapsing–remitting disease, they also exhibit more brain/spinal cord atrophy and T2 lesions in the spinal cord (Antel et al., 2012; Hawker, 2011). The neurodegenerative process that occurs in PPMS appears to spread across connected structures in the brain while proceeding independently in the spinal cord (Rovaris et al., 2008; Ruggieri et al., 2015). However, evidence of gadolinium‐enhanced lesions in PPMS patients also suggested the existence of active inflammation (Ziemssen et al., 2015).

As demyelination and axonal loss translate into the accumulation of neurological disability, certain MRI findings may play a role as disease progression markers. Changes in lesion number or brain and spinal cord volumes seem to correlate with the degree of disability in PPMS patients (Popescu et al., 2013; Rovaris et al., 2008; Stevenson et al., 2004; Ukkonen et al., 2003). They also exhibit abnormalities in white and gray matter, though more pronounced gray matter changes were suggested (Rovaris et al., 2008; Sastre‐Garriga et al., 2005). In addition, gray matter atrophy seems to be a regional phenomenon that occurs at different rates across the brain (Eshaghi et al., 2014; Sepulcre et al., 2006). Regions with an active metabolism, more interconnections with other brain areas or potentially affected by meningeal inflammation may be more prone to atrophy and their volume loss associated with disability worsening (Eshaghi et al., 2014). However, scant information is still available on the effect of specific brain region volumes in this patient population.

Progressive neurological degeneration of PPMS may also affect cognitive functioning, leading to a more frequent and severe impairment than the relapsing–remitting course (Jonkman et al., 2015; Planche et al., 2016). Cognitive performance in PPMS patients seems to be associated with brain volumes, white/gray matter volumes, disease lesions, and certain brain structures (Jonkman et al., 2015; Tur, Penny, et al., 2011). Accumulation of T2 lesions and the consequent exhaustion of frontal lobe plasticity might also contribute to cognitive impairment in these patients (Rocca et al., 2010). Although some brain areas could be particularly involved in neuropsychological test performance, no specific region was clearly identified as a cognitive impairment predictor.

In light of the above, we further assessed brain volumes of specific regions of PPMS patients and their relationship with disability progression and cognitive function over one year.

## 2 METHODS

### 2.1 Study design and participants

This was an analysis of MRI scans from the prospective Understanding Primary Progressive Multiple Sclerosis (UPPMS) cohort study, which was conducted in the Neurology departments of 11 Spanish hospitals. The study was performed according to Good Pharmacoepidemiology Practices, the World Medical Association Declaration of Helsinki, all its amendments, and national regulations. It was approved by the ethics committee of Hospital Universitario 12 de Octubre (Madrid, Spain), and all patients gave their written informed consent.

The study population included 43 patients aged ≥ 18 years, diagnosed with PPMS according to 2010 McDonald criteria, (Polman et al., 2011) and within ten years from its neurologic symptom onset. Patients must have had an MRI scan within the three months previous to their inclusion into the study and no disease‐modifying treatment within the past six months. Participation in any clinical trial and any medical condition that prevented adequate diagnostic evaluation were exclusion criteria.

### 2.2 Assessments

Clinical and MRI data were collected at patient enrollment (baseline) and after 12 months. These data included demographics, medical history of multiple sclerosis, MRI findings, neurological disability assessments, and cognitive function evaluations.

The baseline MRI scan was performed within the three months previous to enrollment as per inclusion criteria. When the investigator did not plan to conduct a 12‐month MRI scan due to clinical reasons, it could be conducted for study purpose following clinical practice procedures and provided that the patient agreed. The following acquisition parameters were recommended: 1.5‐3T, slice thickness < 2 mm, repetition time 9.7 ms, Echo time 4 ms, inversion time 20 ms, and acquisition T2‐FLAIR. A copy of MRI scans was anonymized and sent to Brain Dynamics S.L. (Málaga, Spain) for central volume assessment through an online platform (BD‐Neuroimaging Platform), which was designed to use images acquired from a wide range of machines and used a model for tissue classification achieving an accuracy of 93% (Feng et al., 2014). Every image was assessed under an individualized quality control system, based on the NiftyReg Library applied to the International Consortium for Brain Mapping (ICBM) atlas in the space MNI152. The system converted from DICOM to a more processable format and then applied inhomogeneity correction and intensity normalization techniques to reach the best 3D reconstruction, segmentation, and data extraction. Normalization of T1 images in MNI space and bias field correction were done according to (Tustison et al., 2010), tissue segmentation (white and gray matter) as per (Avants et al., 2011), and multi‐atlas anatomical brain parcellation and labeling for data extraction as per (Wang et al., 2013). T1‐weighted images were used to measure brain volume changes, including the computation of 252 anatomic brain region volumes, and fluid‐attenuated inversion recovery (FLAIR) images were used to locate and quantify brain lesions, thus avoiding the incorrect classification of lesion volumes as gray matter volumes (Chard et al., 2010). Hyperintense regions in T2‐FLAIR images were assessed as lesion burden estimation according to parameters established in the literature (Ong et al., 2012). T1, FLAIR, and proton density sequences were needed to assess black holes. Brain volume changes were measured considering the whole brain, gray and white matter, and each anatomic brain region. Copies of MRI scans were also sent to Hospital Universitari I Politècnic La Fe (Valencia, Spain), where an experienced neuroradiologist (JCP) quantified the number of new or enlarged T2 lesions.

Disability progression was assessed according to the Expanded Disability Status Scale (EDSS; ≥1‐point increase in patients with a baseline score ≤ 5.0 or ≥ 0.5 points in those with a baseline score ≥ 5.5 and confirmed ≥ 3 months later), 9‐Hole Peg Test (9‐HPT; ≥20% increase from baseline and confirmed ≥ 3 months later), and/or Timed 25‐Foot Walk (T25‐FW; ≥20% increase from baseline and confirmed ≥ 3 months later; Lublin et al., 2016).

The Brief Repeatable Neuropsychological Battery (BRNB) was used to assess patient cognitive function (Boringa et al., 2001). It included the Selective Reminding Test (SRT), which provided information on long‐term storage (LTS), consistent long‐term retrieval (CLTR), and selective reminding test‐delayed recall (SRT‐D), along with the Spatial Recall Test (SPART) and delayed recall (SPART‐D), Symbol Digit Modalities Test (SDMT), Paced Auditory Serial Addition Test at a 3‐s rate (PASAT3) and 2‐s rate (PASAT2), and Word List Generation (WLG). Higher scores on these scales indicated a better cognition performance.

### 2.3 Statistical analysis

The number of new/enlarged MRI lesions was described, and brain volume changes from baseline to month 12 were analyzed using Wilcoxon or *t* tests. The relationship of baseline and month‐12 volumes with disability progression was analyzed using R language to perform a Kruskal–Wallis hypothesis test, estimating the difference between patients with and without progression and the optimal cutoff. Mean values and 95% confidence intervals were calculated using the bootstrapping technique, supporting the robustness of the analysis. Furthermore, baseline volumes and changes after 12 months were correlated with BRBN scores throughout the study using Spearman's rank correlations. Correction for multiple comparisons was performed when assessing relationships/correlations in this study.

Missing data were not considered in the analyses, and a significance level of 0.05 was used for statistical testing. The statistical analyses were performed using the Statistical Package for the Social Sciences version 17.0 (SPSS Inc,) and R version 3.5.1 (R Foundation for Statistical Computing).

## 3 RESULTS

### 3.1 Patient characteristics

Forty‐three of the 55 patients enrolled in the UPPMS study between January and July 2017 had MRI scans available for brain volume assessment. Their mean age was 55.7 ± 9.5 years and 65.1% were male (Table 1). The mean time since multiple sclerosis diagnosis and EDSS score were 4.8 ± 5.4 years and 5.1 ± 1.6, respectively.

**TABLE 1:** Baseline patient characteristics (*N* = 43)

Patient characteristics	Value
Age (years), mean ± *SD*	55.7 ± 9.5
Sex, *n* (%)	
Male	28 (65.1)
Female	15 (34.9)
Time since multiple sclerosis diagnosis (years), mean ± *SD*	4.8 ± 5.4
EDSS score, mean ± *SD*	5.1 ± 1.6
Relapses in the previous year, *n* (%)	1 (2.3)^a^

Note

Abbreviations: EDSS, Expanded Disability Status Scale; *SD*, standard deviation.

^a^ This patient experienced a relapse in the previous year with gait involvement.

### 3.2 MRI assessment

**FIGURE 1:**
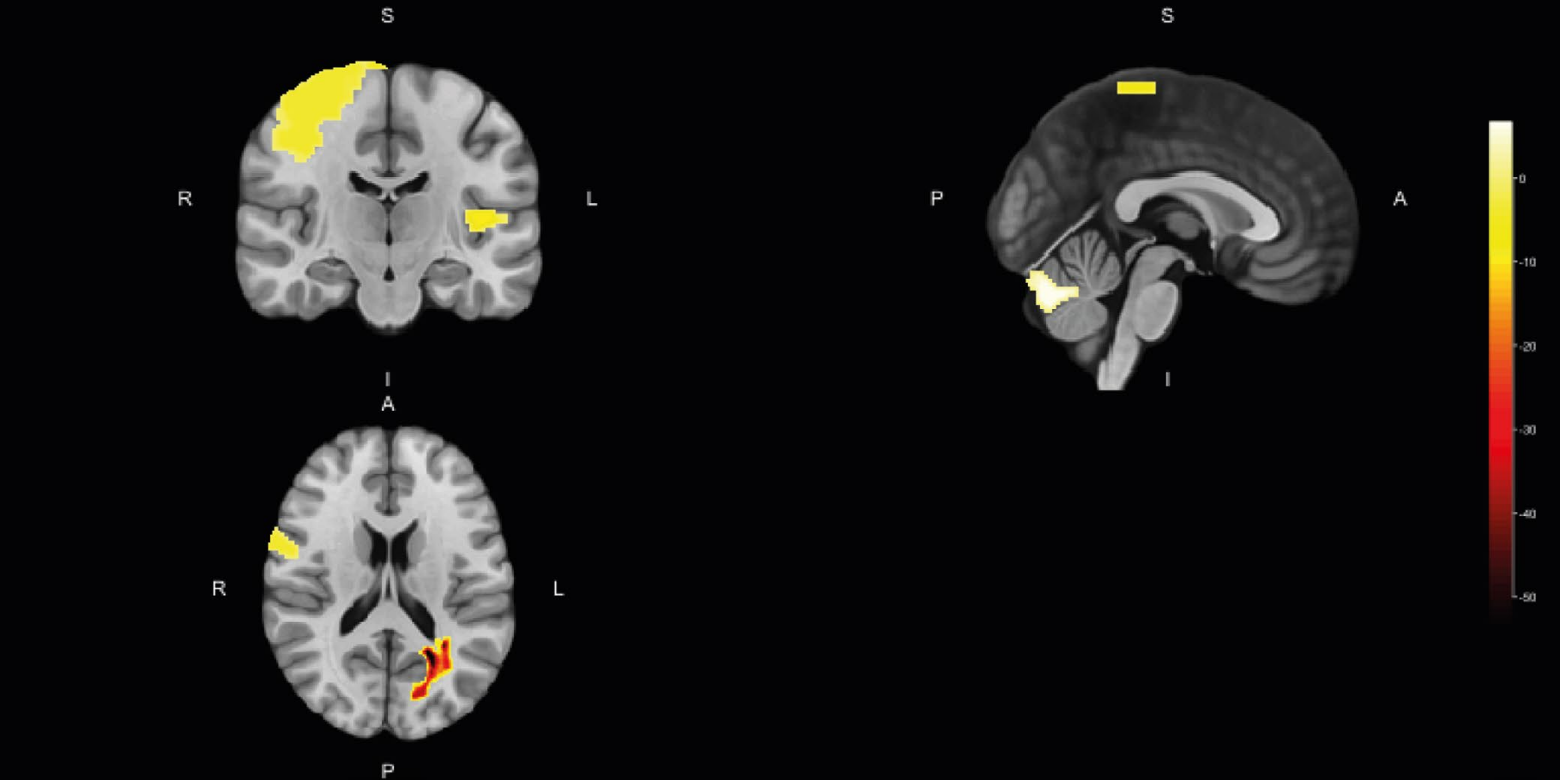
Location of main volumetric differences from baseline to month 12. A, anterior; I, inferior; L, left; P, posterior; R, right; S, superior

Ten patients showed a mean of 1.8 ± 1.3 new/enlarged MRI lesions at month 12. Whole brain, gray matter, and white matter volumes did not change significantly, with mean absolute changes of −2596.8 ± 54,612.5, −795.4 ± 35,930.0, and −1801.3 ± 21,810.4 mm^3^, respectively. However, significant decreases from baseline to month 12 were shown in volumes of white matter adjacent to left straight gyrus, gray matter declive, white matter adjacent to right calcarine sulcus, and white matter adjacent to right inferior occipital gyrus, with mean absolute changes of −94.8 ± 251.2, −80.6 ± 298.3, −3197.7 ± 56,162.6, and −248.6 ± 944.4 mm^3^, respectively (Table 2). Significant increases were also found in volumes of gray matter of left transversal temporal gyrus, white matter adjacent to left middle temporal gyrus (pole), gray matter of right precentral gyrus, and white matter of right inferior fronto‐occipital fasciculus, with mean absolute changes of 53.6 ± 152.3, 32.9 ± 99.9, 226.4 ± 739.4, and 71.9 ± 537.0 mm^3^, respectively (Table 2). Figure 1 depicts the location of main volumetric differences.

**TABLE 2:** Significant changes in MRI scans from baseline to month 12

Volume (mm^3^)	Baseline Mean (95% CI)	Month 12 Mean (95% CI)	*p*
WM adjacent to left straight gyrus	1,032.6 (959–1120)	934.0 (868–1040)	.006
GM declive (V7)	1,192.0 (1130–1310)	1,106.3 (1020–1240)	.016
WM adjacent to right calcarine sulcus	20,399.9 (18239–22560)	17,157.5 (15347–18943)	.018
WM adjacent to right inferior occipital gyrus	2,290.3 (2050–2630)	2042.7 (1860–2330)	.049
GM left transversal temporal gyrus	786.8 (719–872)	839.4 (771–918)	.031
WM adjacent to left middle temporal gyrus (pole)	288.0 (252–315)	321.6 (280–350)	.045
GM right precentral gyrus	9,756.1 (9460–10200)	10,032.2 (9810–10600)	.046
WM right inferior fronto‐occipital fasciculus	3,944.3 (3770–4420)	4,023.2 (3750–4390)	.047

Note

Abbreviations: CI, confidence interval; GM, gray matter; WM, white matter.

### 3.3 Disability progression

**FIGURE 2:**
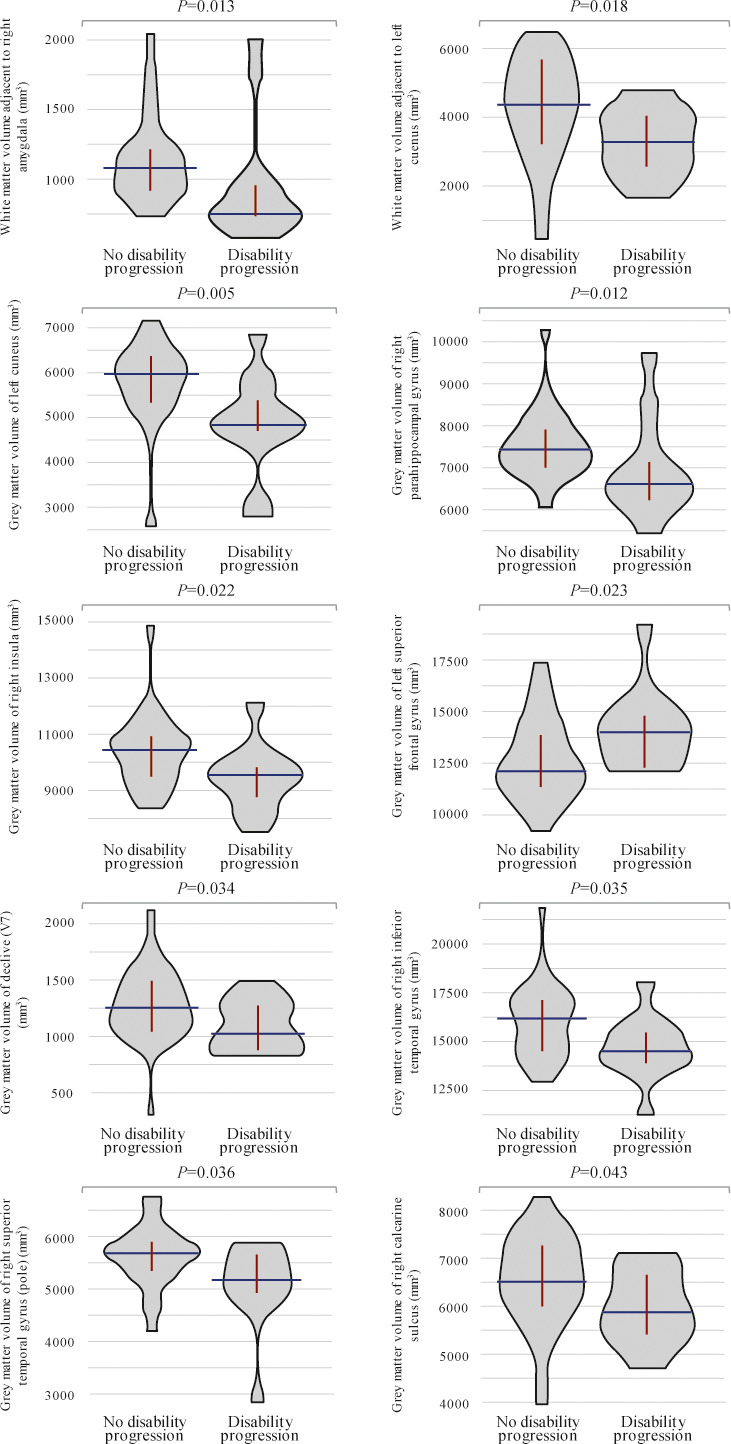
Violin plots showing significant differences in baseline brain region volumes between patients with and without disability progression

**FIGURE 3:**
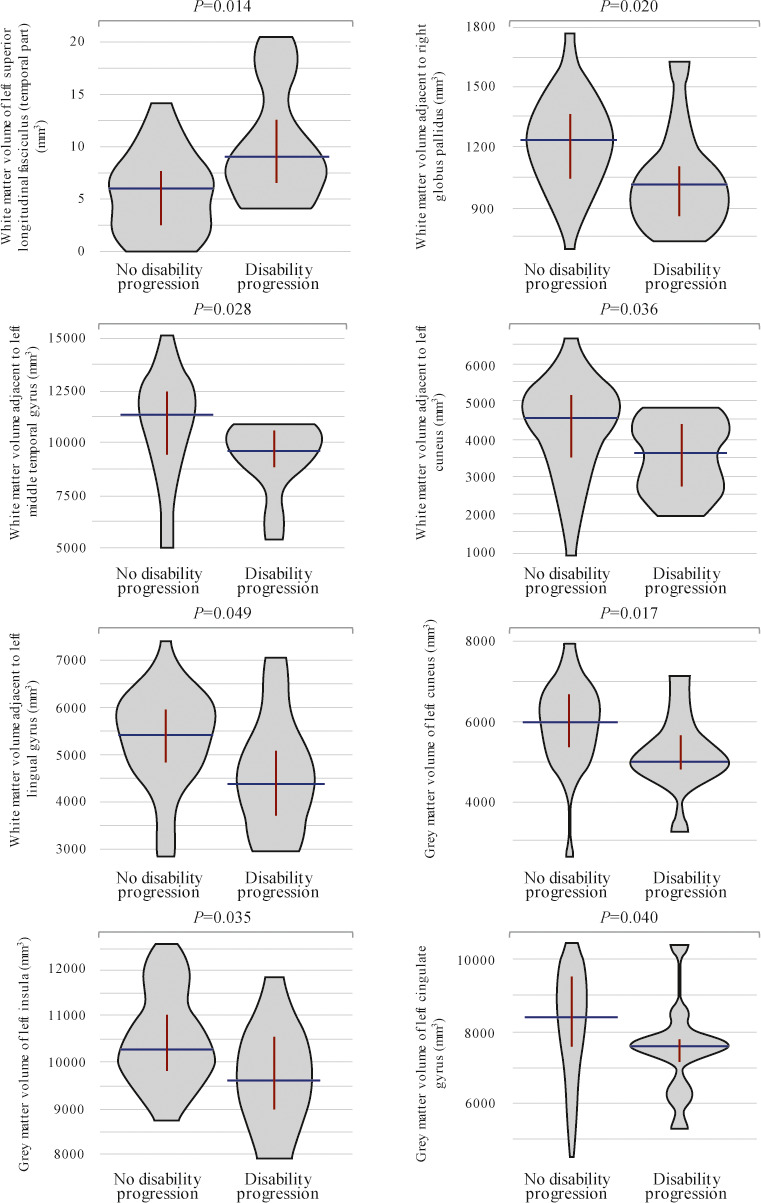
Violin plots showing significant differences in 12‐month brain region volumes between patients with and without disability progression

**FIGURE 4:**
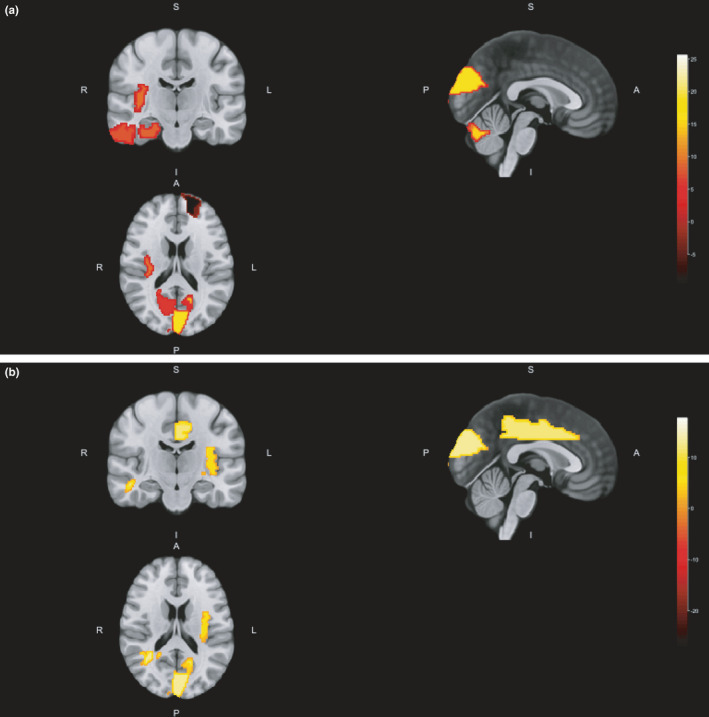
Location of main volumetric differences between patients with and without disability progression at baseline (a) and month 12 (b). A, anterior; I, inferior; L, left; P, posterior; R, right; S, superior

Fifteen (34.9%) patients showed disability progression over the 1‐year study follow‐up. Baseline volumes of white matter adjacent to right amygdala and left cuneus significantly differed between patients with and without disability progression, as well as baseline gray matter volumes of left cuneus, right parahippocampal gyrus, right insula, left superior frontal gyrus, declive, right inferior temporal gyrus, right superior temporal gyrus (pole), and right calcarine sulcus (Table 3; Figure 2). Patients with baseline volumes below cutoffs described in Table 3 were at higher risk of disability progression after one year, except for gray matter volume of left superior frontal gyrus which was higher in patients who progressed. Moreover, 12‐month volumes of white matter of left superior longitudinal fasciculus (temporal part) and adjacent to right globus pallidus, left middle temporal gyrus, left cuneus, and left lingual gyrus also differed between patients with and without disability progression, as well as gray matter volumes of left cuneus, left insula, and left cingulate gyrus (Table 3; Figure 3). Figure 4 depicts main volumetric differences at baseline and month 12 between patients with and without disability progression. Normalized volumetric differences were secondarily analyzed and shown in Table 4.

**TABLE 3:** Significant volumetric differences according to disability progression and optimal cutoffs

Variable	Disability progression Mean (95% CI)	No disability progression Mean (95% CI)	*p*	Cutoff				
				Value	Accuracy	Sensitivity	Specificity	AUC
Baseline volume (mm^3^)								
WM adjacent to right amygdala	896.3 (842.7–1037.5)	1,103.6 (1,116.9–1194.1)	.013	1,364.3	0.364	0.833	0.188	0.747
WM adjacent to left cuneus	3,245.0 (2,843.8–3513.0)	4,367.4 (4,053.2–4302.0)	.018	2,740.0	0.727	0.333	0.875	0.734
GM left cuneus	4,888.1 (4,733.0–5579.4)	5,868.9 (5,475.5–5853.6)	.005	5,414.8	0.682	0.750	0.656	0.779
GM right parahippocampal gyrus	6,797.9 (6,579.3–7215.1)	7,468.5 (7,457.5–7824.7)	.012	7,926.6	0.419	0.833	0.258	0.750
GM right insula	9,321.1 (9,480.4–9673.8)	10,270.3 (10,240.2–10390.1)	.022	11,009.1	0.372	0.909	0.188	0.733
GM left superior frontal gyrus	13,781.0 (13,923.6–14717.3)	12,532.4 (12,250.5–12725.8)	.023	14,623.1	0.682	0.250	0.844	0.724
GM declive (V7)	1,073.0 (952.5–1189.1)	1,261.9 (1,210.0–1348.8)	.034	1,143.7	0.636	0.583	0.656	0.711
GM right inferior temporal gyrus	14,595.4 (14,291.9–15619.2)	15,827.6 (15,773.6–16413.2)	.035	15,705.3	0.636	0.833	0.563	0.708
GM right superior temporal gyrus (pole)	5,246.1 (4,838.0–5369.0)	5,644.3 (5,558.5–5685.2)	.036	5,213.0	0.738	0.500	0.833	0.711
GM right calcarine sulcus	6,045.1 (5,828.6–6306.2)	6,620.6 (6,558.2–6830.8)	.043	5,933.0	0.705	0.500	0.781	0.701
12‐month volume (mm^3^)								
WM left superior longitudinal fasciculus (temporal part)	9.6 (9.7–10.3)	5.3 (3.8–6.3)	.014	6.2	0.628	0.750	0.581	0.743
WM adjacent to right globus pallidus	1,006.6 (985.9–1111.5)	1,214.9 (1,196.3–1278.2)	.020	1,205.5	0.595	0.818	0.516	0.739
WM adjacent to left middle temporal gyrus	9,584.6 (9,038.5–10033.4)	11,058.5 (10,101.8–11276.7)	.028	8,551.9	0.636	0.167	0.813	0.716
WM adjacent to left cuneus	3,556.0 (3,308.1–4037.0)	4,326.8 (4,055.1–4555.0)	.036	3,103.5	0.698	0.417	0.806	0.710
WM adjacent to left lingual gyrus	4,473.1 (4,241.1–4911.8)	5,394.8 (5,073.6–5246.9)	.049	5,267.1	0.636	0.833	0.563	0.695
GM left cuneus	5,200.2 (5,012.9–5572.0)	5,971.1 (5,727.6–6095.6)	.017	6,140.8	0.568	0.833	0.469	0.734
GM left insula	9,644.3 (9,508.9–10104.6)	10,478.2 (10,119.9–10682.5)	.035	10,905.0	0.455	0.917	0.281	0.708
GM left cingulate gyrus	7,403.9 (7,670.2–8199.0)	8,380.3 (7,942.1–8272.5)	.040	7,758.4	0.727	0.750	0.719	0.703

Note

Abbreviations: AUC, area under the curve; CI, confidence interval; GM, gray matter; WM, white matter.

**TABLE 4:** Significant normalized volumetric differences according to disability progression and optimal cutoffs

Variable	Disability progression Mean (95% CI)	No disability progression Mean (95% CI)	*P*	Cutoff				
				Value	Accuracy	Sensitivity	Specificity	AUC
Baseline volume (mm^3^)								
WM adjacent to right amygdala	0.1 (0.0–0.1)	0.1 (0.1–0.1)	.001	0.1	0.738	0.727	0.742	0.824
WM adjacent to left cuneus	0.2 (0.2–0.3)	0.3 (0.3–0.3)	.023	0.3	0.682	0.750	0.656	0.724
GM left cuneus	0.3 (0.3–0.4)	0.4 (0.4–0.4)	.021	0.4	0.705	0.667	0.719	0.727
GM right parahippocampal gyrus	0.5 (0.4–0.5)	0.5 (0.5–0.5)	.006	0.5	0.780	0.727	0.800	0.776
GM right insula	0.7 (0.6–0.7)	0.7 (0.7–0.8)	.034	0.7	0.674	0.545	0.719	0.716
GM left superior frontal gyrus	1.0 (0.9–1.0)	0.9 (0.8–0.9)	.020	0.9	0.651	0.727	0.625	0.736
GM right inferior temporal gyrus	1.0 (1.0–1.1)	1.1 (1.1–1.2)	.049	1.1	0.568	0.500	0.594	0.695
GM right culmen (C4−5)	0.5 (0.4–0.5)	0.4 (0.4–0.4)	.033	0.5	0.682	0.667	0.688	0.711
12‐month volume (mm^3^)								
WM left cingulum (cingulate gyrus)	0.0 (0.0–0.1)	0.1 (0.1–0.1)	.049	0.1	0.614	0.750	0.563	0.695
WM adjacent to right putamen	0.3 (0.2–0.3)	0.3 (0.3–0.3)	.043	0.3	0.705	0.667	0.719	0.701
GM left cuneus	0.4 (0.3–0.4)	0.4 (0.4–0.4)	.014	0.4	0.705	0.667	0.719	0.740
GM left posterior cingulate gyrus	0.2 (0.1–0.2)	0.1 (0.1–0.2)	.041	0.2	0.738	0.545	0.806	0.710
GM triangular part of right inferior frontal gyrus	0.5 (0.4–0.5)	0.4 (0.4–0.5)	.037	0.4	0.581	0.667	0.548	0.707
GM left hippocampus	0.4 (0.3–0.4)	0.4 (0.4–0.4)	.009	0.4	0.791	0.636	0.844	0.764
GM right culmen (C4−5)	0.5 (0.4–0.5)	0.4 (0.4–0.5)	.025	0.5	0.659	0.583	0.688	0.721

Note

Abbreviations: AUC, area under the curve; CI, confidence interval; GM, gray matter; WM, white matter.

### 3.4 Cognition performance

**FIGURE 5:**
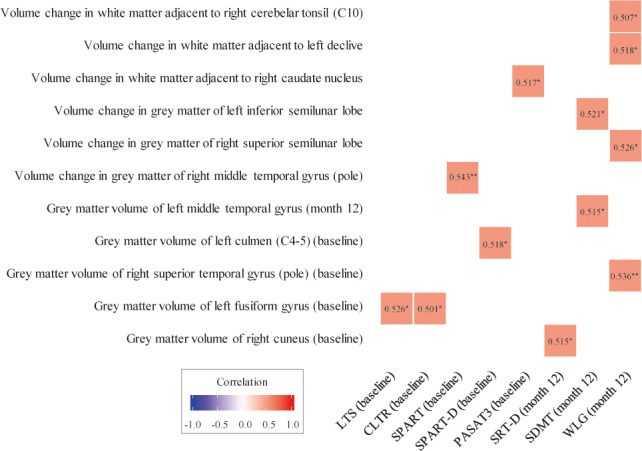
Correlation between MRI findings and cognitive function performance. Correlations with rho correlation coefficient > 0.4 are presented. *p < .05; **p < .01. CLTR, Consistent Long‐Term Retrieval; LTS, Long‐Term Storage; PASAT3, Paced Auditory Serial Addition Test 3‐Second Trial; SDMT, Symbol Digit Modalities Test; SPART, Spatial Recall Test; SPART‐D, Spatial Recall Test‐delayed recall; SRT‐D, Selective Reminding Test‐Delayed Recall; WLG, Word List Generation

**FIGURE 6:**
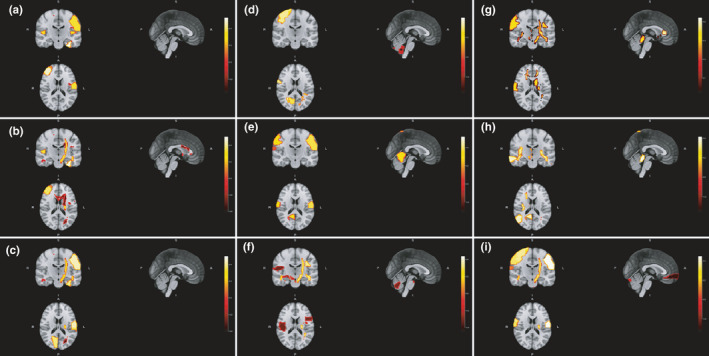
Location of main baseline region volumes correlated with baseline scores on Long‐Term Storage (a), Consistent Long‐Term Retrieval (b), Selective Reminding Test‐Delayed Recall (c), Spatial Recall Test (d), Spatial Recall Test‐Delayed (e), Symbol Digit Modalities Test (f), Paced Auditory Serial Addition Test 3‐Second Trial (g), Paced Auditory Serial Addition Test 2‐Second Trial (h) and Word List Generation (i). A, anterior; I, inferior; L, left; P, posterior; R, right; S, superior

**FIGURE 7:**
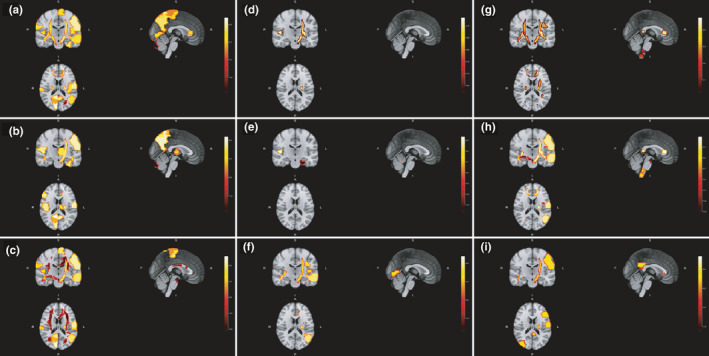
Location of main baseline region volumes correlated with 12‐month scores on Long‐Term Storage (a), Consistent Long‐Term Retrieval (b), Selective Reminding Test‐Delayed Recall (c), Spatial Recall Test (d), Spatial Recall Test‐Delayed (e), Symbol Digit Modalities Test (f), Paced Auditory Serial Addition Test 3‐Second Trial (g), Paced Auditory Serial Addition Test 2‐Second Trial (h) and Word List Generation (i). A, anterior; I, inferior; L, left; P, posterior; R, right; S, superior

Cognitive performance over the study is summarized in Table 5. Baseline gray matter volumes of right cuneus and right superior temporal gyrus (pole) positively correlated with SRT‐D and WLG performance after the 1‐year follow‐up, respectively (Figure 5). Volume changes from baseline to month 12 in gray matter of right superior semilunar lobe, white matter adjacent to left declive, and white matter adjacent to right cerebellar tonsil (C10) were also positively correlated with WLG scores after one year (Figure 5). Moreover, the change in gray matter volume of left inferior semilunar lobe positively correlated with SDMT performance at study end (Figure 5). Other correlations with rho coefficient > 0.4 are shown in Figure 5. Figures 6 and 7 depict main region volumes correlated with cognitive function.

**TABLE 5:** Overview of cognitive performance (*N* = 43)

Neuropsychological battery scores, mean ± *SD*	Baseline	Month 12
LTS	39.4 ± 18.0	48.3 ± 14.6
CLTR	29.6 ± 17.2	40.2 ± 16.8
SRT‐D	7.7 ± 2.6	9.3 ± 2.6
SPART	14.2 ± 5.3	16.7 ± 5.5
SPART‐D	5.1 ± 2.4	5.6 ± 2.5
SDMT	29.2 ± 12.9	30.7 ± 12.4
PASAT3	33.9 ± 13.3^a^	38.2 ± 15.2^c^
PASAT2	26.9 ± 10.5^b^	28.0 ± 12.1^c^
WLG	20.4 ± 6.4	21.5 ± 6.2

Note

Abbreviations: CLTR, Consistent Long‐Term Retrieval; LTS, Long‐Term Storage; PASAT2, Paced Auditory Serial Addition Test 2 Second Trial; PASAT3, Paced Auditory Serial Addition Test 3 Second Trial; *SD*, standard deviation; SDMT, Symbol Digit Modality Test; SPART, Spatial Recall Test; SPART‐D, Spatial Recall Test‐Delayed; SRT‐D, Selective Reminding Test‐Delayed Recall; WLG, Word List Generation.

^a^ Missing data, *n* = 2.

^b^ Missing data, *n* = 5.

^c^ Missing data, *n* = 1.

## 4 DISCUSSION

This MRI analysis from the UPPMS cohort study showed decreasing volumes of gray matter declive and white matter adjacent to left straight gyrus, right calcarine sulcus, and right inferior occipital gyrus over one year. Conversely, gray matter volumes of left transversal temporal gyrus and right precentral gyrus increased, as well as white matter volumes of right inferior fronto‐occipital fasciculus and adjacent to left middle temporal gyrus (pole). Although multiple sclerosis has been considered a predominantly white matter disease due to its demyelinating pathophysiology, gray matter involvement has also been recognized (Calabrese et al., 2015). Patterns of volume loss differ according to the clinical course of multiple sclerosis, with predominant atrophy around the cerebral ventricles in relapsing–remitting patients and cortical/subcortical regions in those with progressive disease (Pagani et al., 2005). Evidence on PPMS supports a widespread supratentorial and infratentorial tissue loss, including the gray matter of deep (basal ganglia, thalamus, and claustrum) and cortical regions (insular cortex and superior/inferior frontal, pre‐/‐central, posterior cingulated, parahippocampal, supramarginal, superior temporal, middle occipital, and inferior occipital gyri; Tavazzi et al., 2015). Decreasing volumes of several deep gray matter regions (putamen, caudate, and thalamus) and some cortical or infratentorial areas (limbic, occipital, frontal and parietal lobes, and cerebellum) may be evident after one year (Sepulcre et al., 2006). The rate of volume loss affects different structures at variable rates, with the fastest involvement of cingulate gyri, adjacent precuneus, cerebellum, precentral gyri, thalami and insula in comparison with healthy controls over five years (Eshaghi et al., 2014). Several mechanisms have been proposed to underlie this neurodegeneration, such as immune‐related, mitochondrial injury, or retrograde degeneration due to white matter damage (Calabrese et al., 2015). However, it is noteworthy that volume measurements can also be affected by other factors such as inflammation or edema (Cortese et al., 2019).

Neurological changes of PPMS need to be monitored due to their impact on patient health and daily activities. Indeed, our MRI analysis supports the relationship of brain region volumes with disability progression and cognitive performance over one year. Specifically, baseline volumes of white matter adjacent to right amygdala and left cuneus could predict disability progression, as well as baseline gray matter volumes of left cuneus, left superior frontal gyrus, right parahippocampal gyrus, right insula, right inferior temporal gyrus, right superior temporal gyrus (pole), right calcarine sulcus, and declive. In addition, patients with and without disability progression differed in their 12‐month white matter volumes of left superior longitudinal fasciculus (temporal part) and adjacent to left middle temporal gyrus, left cuneus, left lingual gyrus, and right globus pallidus, as well as gray matter volumes of left cuneus, left insula, and left cingulate gyrus. Eshaghi et al. (2014) reported the association of volume loss in cingulate gyrus with 5‐year clinical disability according to the Multiple Sclerosis Functional Composite in PPMS patients, without a significant association between the rate of gray matter volume loss and changes in EDSS scores or T2 lesion accrual per annum (Eshaghi et al., 2014). The contribution of cingulate region atrophy to this clinical disability was then attributed to motor control via motor cortex and spinal cord connections (Eshaghi et al., 2014). Gray matter atrophy in the right sensory‐motor cortex of PPMS patients was also associated with greater upper limb disability according to 9‐HPT, despite lacking association between gray matter damage and EDSS scores (Bodini et al., 2009). Another study found, however, that atrophy in the right middle frontal gyrus, right lateral fissure, left angular gyrus, and prepontine cistern correlated with changes in EDSS scores and T1 lesion volumes after 15 months (Pagani et al., 2005). In addition, the disability resulting from PPMS may be affected by demyelination and axonal loss in normal‐appearing white and gray matter, (Bodini et al., 2009; Ramio‐Torrenta et al., 2006; Rovaris et al., 2008; Tur, Khaleeli, et al., 2011) leading to suggest the combination of both white and gray matter damage to more accurately predict disability accrual (Tur, Khaleeli, et al., 2011). Furthermore, brain and spinal cord atrophy can provide complementary information, as microstructural changes, lower cross‐sectional cord area, and volume loss also contribute to neurological disability (Cortese et al., 2020; Rovaris et al., 2008; Tsagkas et al., 2019). Although these studies offer insights into the implications of neurological changes in PPMS disability, comparisons cannot be performed due to methodological differences and further information is still needed to confirm the role of each specific region.

With regard to cognition, our findings support that baseline volumes of certain regions such as gray matter of right cuneus and right superior temporal gyrus could predict 1‐year cognitive performance according to WLG and SRT‐D scores, respectively. Similarly, volume changes in gray matter of right superior semilunar lobe and white matter adjacent to left declive and right cerebellar tonsil also correlated with WLG scores, and gray matter volume change in left inferior semilunar lobe correlated with SDMT performance after one year. Changes in human connectome might translate into brain volume changes that affect specific function performance (Charalambous et al., 2019). Cognitive impairment in multiple sclerosis results from a complex interplay of factors such as premorbid cognitive status, lesional/nonlesional tissue damage, and adaptative/maladaptive functional reorganization (Jonkman et al., 2015). MRI measures of white and gray matter injury have been reported as contributors to cognitive status across different multiple sclerosis types, with a dominant role of subcortical gray matter injury in PPMS patients (Jonkman et al., 2015). Other studies also suggested that cognitive impairment in these patients went beyond white matter damage, pointing at gray matter loss as the primary contributor (Gouveia et al., 2017; Tur, Penny, et al., 2011). Both neocortical and subcortical gray matter were involved in cognitive performance, though subcortical gray matter played a major role in information processing speed and verbal/visuospatial learning and memory (Gouveia et al., 2017). Gray matter loss in the thalamus was reported as a correlate of this cognitive impairment, (Gouveia et al., 2017) as well as other areas such as right superior temporal gyrus or anterior cingulate cortex (Riccitelli et al., 2011). In addition, functional MRI assessment showed higher activation of the cerebellum and cortical regions such as the insula or sensory‐motor cortex in cognitively impaired PPMS patients, compensating the lower activation of other areas involved in working memory in a variable network recruitment process over time (Rocca et al., 2010). Furthermore, lesion burden seems to be involved in verbal memory (Tur, Penny, et al., 2011) and attention/speed of information processing (Penny et al., 2010) and might contribute to exhaustion of frontal lobe plasticity, (Rocca et al., 2010) and normal‐appearing white or gray matter volume was associated with attention/speed of visual information (Penny et al., 2010; Tur, Penny, et al., 2011) and executive function performance, (Penny et al., 2010; Ramio‐Torrenta et al., 2006) which underscore the complexity of neurological damage in cognitive disfunction of PPMS patients.

We acknowledge that the study has limitations that should be considered, including its uncontrolled design, relatively small sample size, short‐term follow‐up and absence of spinal cord evaluation, and uniform procedures when conducting the MRI scans. However, it provides longitudinal data on a large number of brain region volumes and their relationship with neurological and cognitive function in PPMS patients. Moreover, its prospective multicentre nature and the fact that the MRI scans were performed according to routine procedures of 11 participating sites favors the generalizability of our findings.

In conclusion, our MRI analysis provides a detailed description of region volume changes exhibited by PPMS patients over one year, as well as proposing specific white/gray matter region volumes as potential predictors of disability progression and cognitive performance. These include gray matter volume of right cuneus or right superior temporal gyrus as cognitive predictors, and white matter volume adjacent to right amygdala or left cuneus and gray matter volume of left cuneus, left superior frontal gyrus, right parahippocampal gyrus, right insula, right inferior temporal gyrus, right superior temporal gyrus (pole), right calcarine sulcus, or declive as disability progression predictors. However, further studies are needed to confirm our findings and verify their usefulness in clinical practice.

## CONFLICT OF INTEREST

The authors of this manuscript declare that Daniel Prefasi and Jorge Maurino are employees of Roche Farma S.A. Francisco Pérez‐Miralles was part of the steering committee of the UPPMS study and has received compensation for serving on scientific advisory boards or speaking honoraria from Almirall, Biogen Idec, Genzyme, Merck‐Serono, Mylan, Novartis, Roche, Sanofi‐Aventis, and Teva, outside the submitted work. Antonio García‐Merino has received consultant and/or lecture fees from Merck, Teva, Biogen, Novartis, Roche, and Sanofi. The remaining authors declare no conflict of interest to disclose.

## AUTHOR CONTRIBUTION

All authors had complete access to all study data and assume complete responsibility for the integrity of the data and accuracy of the data analysis. FPM conceived and designed the study, recruited and completed the evaluations of the participants, led statistical analysis, wrote the first draft of the manuscript, and revised the draft. DP conceived and designed the study, led statistical analysis, wrote the first draft, and revised the draft. AGM, JRA, GI, VML, FGG, MLMG, LRT, LCF, OF, and SMG recruited and completed the evaluations of the participants and revised the draft. JM conceived and designed the study and revised the draft. JCP collected the MRI data. BC assessed in the study design and revised the draft. All authors have read and approved the final manuscript.

### Peer Review

The peer review history for this article is available at https://publons.com/publon/10.1002/brb3.2044.

## Data Availability

Qualified researchers may request access to individual patient‐level data through the clinical study data request platform (www.clinicalstudydatarequest.com). Further details on Roche's criteria for eligible studies are available here (https://clinicalstudydatarequest.com/Study‐Sponsors/Study‐Sponsors‐Roche.aspx). For further details on Roche's Global Policy on the Sharing of Clinical Information and how to request access to related clinical study documents, see here (https://www.roche.com/research_and_development/who_we_are_how_we_work/clinical_trials/our_commitment_to_data_sharing.htm).
